# Anticoagulation in Atrial Fibrillation Associated With Cardiac Amyloidosis: A Narrative Review

**DOI:** 10.7759/cureus.61557

**Published:** 2024-06-03

**Authors:** Tejbir S Monga, Mfonido Ekong, Kayé Patrick, Tulasi Geethika Bommana Boyena, Aneela Satya Ravanam, Santiago Vargas, Nur Bengisu Mavus, A P Lakshmi, Kanwaraj Singh, Ramya Reshma Vegesna, Kiran Abbas

**Affiliations:** 1 Internal Medicine, Spartan Health Sciences University, Vieux Fort, LCA; 2 Internal Medicine, St. George's University School of Medicine, True Blue, GRD; 3 Anesthesiology, Spanish Town Hospital, Spanish Town, JAM; 4 Internal Medicine, Kingston Public Hospital, Kingston, JAM; 5 Anesthesiology, Government Medical College, Anantapur, IND; 6 Internal Medicine, Universidad de los Andes, Bogota, COL; 7 Medicine, Lokman Hekim University, Ankara, TUR; 8 Cardiology, Caritas Hospital, Kerala, IND; 9 Internal Medicine, Maharishi Markandeshwar Institute of Medical Sciences and Research, Ambala, IND; 10 Internal Medicine, Alluri Sitarama Raju Academy of Medical Sciences, Eluru, IND; 11 Community Health Sciences, Aga Khan University, Karachi, PAK

**Keywords:** atrial fibrillation cardiac amyloidosis, attr amyloidosis, amyloidosis al, anticoagul, atrial fibrillation (af)

## Abstract

Cardiac amyloidosis (CA) involves the abnormal deposition and accumulation of amyloid proteins in the heart muscle. A hallmark of disease progression is declining heart function, which can lead to structural irregularities, arrhythmias, and ultimately heart failure. Atrial fibrillation (AF) is the most common arrhythmia that presents in CA patients, and this arrhythmia is significant because it can moderately increase the risk of patients developing intracardiac thrombi, thereby putting them at risk for thromboembolic events. The management of this complication entails the use of anticoagulants like vitamin K antagonists and direct oral anticoagulants to reduce the risk of thrombus formation. This article seeks to review AF in CA and the use of anticoagulation therapy for the management and reduction of thromboembolic risk. The major conclusions of this review are centered around the need for safe administration of anticoagulant therapy to CA patients, regardless of their CHA2DS2-VASc risk score. This review highlights the importance of taking a multidisciplinary or collaborative approach to CA treatment to ensure that all aspects of this multifaceted disease can be properly managed while minimizing adverse events like bleeding risk and drug-drug interactions.

## Introduction and background

Cardiac amyloidosis (CA) is an uncommon disease characterized by the accumulation of specific precursor protein genes, amyloid fibrils, in the myocardium [1]. CA has a prevalence of 13% in those individuals who are known to have heart failure with preserved ejection fraction (HFpEF) [2]. Two major types of CA exist, including light-chain amyloidosis (AL) and transthyretin amyloidosis (ATTR). The pathophysiology of AL and ATTR involves the deposition of misfolded and overproduced light-chain immunoglobulins and monomers of transthyretin, respectively [1]. The ATTR variant can further be classified as wild-type/non-genetic (ATRwt) or a mutant/hereditary form (ATTRmut).

The myocardial infiltration and subsequent fibrosis cause hypertrophy and impaired ventricular contractility and relaxation. This most commonly leads to the development of restrictive cardiac muscle disease and eventually heart failure, in particular HFpEF. González-López et al., in a prospective study, were able to highlight that wild-type transthyretin amyloidosis (ATTRwt) is a leading cause of HFpEF, affecting 15% of the studied population [2].

CA is also associated with atrial fibrillation (AF). The association between the two varies; however, it is thought to arise due to an elevated atrial afterload, an elevated preload secondary to mitral or tricuspid regurgitation, and increased sympathetic activity [3]. The burden of AF in CA varies with subtype. Generally, arrhythmias are most common in AL, with a prevalence of up to 60% [4]. AF is common in CA and is seen in up to 70% of patients with the ATTRwt subtype [5].

Amyloidosis is a significant determinant of thrombosis due to impaired ventricular dysfunction and subsequent blood stasis [6]. Other mechanisms of thrombogenesis are secondary to the amyloid deposits causing endothelial damage and a hypercoagulable state [3,6]. Additionally, the presence of AF potentiates thromboembolic events in CA patients. A study revealed that 16% of 1100 ATTR patients with AF had either a peripheral embolism, short-term ischemic attack, or stroke, with an incidence of 1.64 embolic events per 100 patients/year (95% CI: 1.2-2.2) [7,8]. There is also a greater likelihood of embolism in patients with AF and CA (37%) than in patients with AF only (19%) [8,9].

Anticoagulation is the mainstay treatment for patients with AF and is especially recommended for patients who have CA, regardless of their CHA2DS2VASc score [10]. It has been highlighted in multiple studies that anticoagulation is safe for stroke prevention, and the incidence of thromboembolic events was increased in nonanticoagulated patients as compared to those who received anticoagulation (4.8 embolism events per 100 patients per year, 95% CI: 2.0-11.6 vs. 1.7 embolism events per 100 patients per year, 95% CI: 1.12.6) [10,11].

The objective of the review is to systematically examine and synthesize existing literature on the management of anticoagulation in patients who have both AF and CA. The review aims to evaluate the efficacy and safety of anticoagulation therapies in managing AF in patients with CA, highlighting specific risks associated with various treatment options.

## Review

Pathophysiology

Although there are several different amyloid disorders, immunoglobulin AL and ATTR are the two primary forms that account for 95% of all CA cases [12]. In AL-CA, the clonal plasma cells produce monoclonal amyloid precursors, while cardiac infiltration by the TTR proteins, which are derived from the liver, causes ATTR-CA. Atria and ventricles are more commonly infiltrated by amyloid fibrils in CA, although they can affect any part of the heart [13]. These deposits cause structural damage, interfere with normal heart function, and can lead to heart failure. It is important to understand the pathophysiology of CA in order to aid in various available therapeutic choices.

The pathogenesis of CA is a dual-impact process that involves amyloid deposition in the cardiac tissues and a direct toxic effect causing oxidative stress and mitochondrial damage by the circulating pre-amyloid proteins [14,15]. AF is the most common form of atrial tachyarrhythmia seen in almost 70% of patients upon diagnosis of CA [16,17]. There are many pro-arrhythmogenic factors that relate to the high incidence of atrial arrhythmias. The toxic inflammatory response caused by the buildup of amyloid fibers in the myocardium results in stiffening of the ventricular walls, leading to impaired relaxation and restrictive filling. This results in an increase in left atrial enlargement, which plays a crucial role in the formation of AF [5]. In atria, myocyte contractility and homogenous electrical conduction are disrupted due to amyloid fibril deposition, giving rise to functional reentry and leading to the development of AF [5]. It also causes significant structural and electrical remodeling, thus elevating the left atrial pressures chronically, leading to dilation, dysfunction with increased ectopic beats arising from pulmonary veins, and ultimately AF [17]. Fibrosis and oxidative stress are the direct harmful effects of amyloid fibrils on atrial cardiomyocytes, and these conditions are also recognized to be important precursors of AF [18]. Specifically, isolated atrial amyloidosis is more prevalent in females and elderly people, and patients having mitral valve replacement surgery, as stated by Röcken et al., are more likely to have AF, which manifests as permanent or persistent AF [8,19].

The effects of amyloid fibrils on ventricular myocytes cause undulated infiltration, microvascular ischemia, and direct cytotoxicity, which leads to ventricular arrhythmias [20]. Reentrant tachycardia is frequently observed in CA after non-sustained ventricular tachycardia and premature ventricular contractions (PVC) [21]. Purkinje system-derived PVC can also result in ventricular fibrillation (VF); however, the precise process by which VF arises in CA is unknown and is linked to a dismal prognosis [22].

The clinical course of systemic amyloidosis is determined by cardiac involvement; nonetheless, extensive myocardial derangements result in cardiac issues [23,24]. The data is limited, but the insoluble protein fibrils themselves lay the groundwork for arrhythmia, causing electrical and structural remodeling that can result in various myocardial arrangements in patients with normal rhythm, AF, or patients receiving anticoagulation for an arrhythmia [25-28].

Atrial myopathy may be a basic contributor to the production of intracardiac thrombi in CA. Regardless of the size of the atrial cavity, diastolic malfunctioning, or electrical atrial cavity, CA was found to be associated with atrial myocardial dysfunction as revealed by strain echocardiography [29,30]. Thus, atrial myopathy may account for the finding of thrombi and embolic events with sinus rhythm or in spite of adequate anticoagulation [25]. The CHA2DS2-VASc score, which represents a proxy for vascular fragility, has been shown to accurately detect the chances of stroke outside the occurrence of AF, even if it was not explicitly designed for non-AF circumstances. This has been found in other cardiac disorders as well [31,32].

Diagnosis and evaluation

The need for CA evaluation arises from clinical suspicion of the disease. The typical presentation of heart failure symptoms like peripheral edema and ascites is common; however, patients could present with nonspecific symptoms such as weakness, fatigue, and weight loss [33,34]. AF could also be the first presentation, but it is often treated in isolation long before CA is even considered. Hence, in a patient presenting with symptoms such as AF, it is important to use distinct cues in an appropriate clinical context to properly evaluate for and potentially diagnose concomitant CA. For example, associated symptoms like bilateral carpal tunnel syndrome, spinal stenosis due to ligamentum flavum infiltration, and the abrupt resolution of hypertension that leads to reduction or discontinuation of antihypertensive therapy can be benchmarks for possible CA [33,34].

Where CA is suspected, an ECG and a two-dimensional echocardiogram can be used in tandem for diagnosis. Findings of low voltage on ECG in the presence of increased ventricular muscle thickness on echo are pathognomonic of CA, and further laboratory studies, advanced imaging like MRI, or cardiac/bone marrow biopsy can then be used to confirm the diagnosis, as the absence of low voltage on ECG does not rule out the disease. Several laboratory markers, including a chronically elevated troponin level in the absence of acute coronary syndrome, an elevated N-terminal pro-B-type natriuretic peptide level, and an abnormal serum free light-chain assay, are useful noninvasive methods of disease confirmation with varying levels of sensitivity. Furthermore, a gadolinium contrast cardiac MRI is about 93% sensitive for CA diagnosis with a diffuse and subendocardial late gadolinium enhancement pattern signifying disease. However, a major limitation of this method is the contraindication of contrast administration in patients with reduced kidney function.

**Figure 1:**
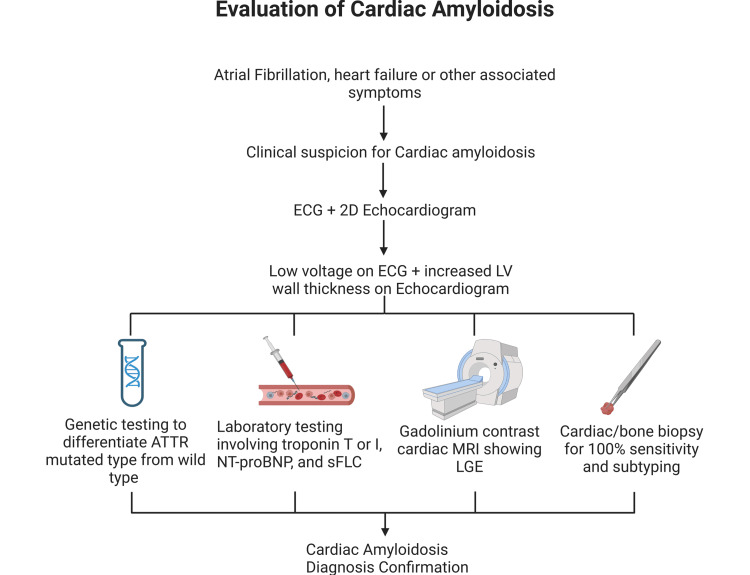
Illustration of the evaluation of CA in suspected patients ATTR, transthyretin amyloidosis; CA, cardiac amyloidosis; LGE, late gadolinium enhancement; LV, left ventricular; NT-proBNP, N-terminal pro-B-type natriuretic peptide; sFLC, serum free light-chain Image credits: Tejbir S. Monga and Mfonido Ekong

Lastly, an endomyocardial biopsy offers 100% sensitivity for CA and is the gold standard for diagnosis confirmation; however, it is the most invasive method and also offers a 1% chance of iatrogenic ventricular perforation. A bone marrow biopsy or genetic testing could also be performed in certain types of CA to differentiate between subtypes (Figure 1) [33].

Current anticoagulation strategies

According to present guidelines, patients with AF should receive anticoagulant therapy depending on the CHA2DS2 VASC score, where an annual stroke risk of 2% is an indication for anticoagulation [35]. However, individuals with CA are at increased risk of developing thrombus and embolic events, particularly in the setting of AF [25]. In a study, a total of 156 patients with CA had a transesophageal echocardiogram (TEE), of which 64% had a history of AF. Results show that 27% had an intracardiac thrombus. A multivariate analysis showed that AF was independently correlated with intracardiac thrombus (OR 6.8, 95% CI: 2.5-20.7, P = 0.0001). This data demonstrated the high incidence of atrial thrombus in this population [36]. Retrospective cohort research involving 100 consecutive ATTR-CA patients who had TEE prior to AF cardioversion. Results indicate no connection between the CHADS-VASc score and LAA thrombus in subjects with ATTR-CA (P = 0.14). Based on these results and the fact that there is atrial dysfunction present in patients with ATTR-CA, the recent guidelines recommend anticoagulation for patients with CA and AF regardless of the CHA2DS2 VASC score and taking into account individualized patient bleeding risk [5,37].

Currently, there are two main therapies for minimizing the risk of stroke in individuals suffering from AF. Vitamin K antagonists (VKAs) were the first pharmacological group used in this scenario. Warfarin, a VKA, inhibits the enzyme epoxide reductase, which is the key enzyme for vitamin K-dependent carboxylation, and therefore affects the formation of coagulation factors (II, VII, IX, X, C, S) [38]. Trials have shown significant evidence of stroke prevention in patients with AF receiving warfarin treatment [39]. However, the onset of direct oral anticoagulants (DOACs) has changed the anticoagulation management of AF. There are four main DOACs. Apixaban, edoxaban, and rivaroxaban act as factor Xa inhibitors. Dabigatran acts as a factor IIa (thrombin) inhibitor [40]. Trials using these four DOACs have shown that they are superior to warfarin in avoiding a stroke or systemic embolism in individuals with AF, except in patients with medium to severe mitral stenosis or mechanical heart valves. They have also shown substantially decreased risks of significant bleeding when in comparison with warfarin [41-44]. Current guidelines only recommend warfarin over DOACs in individuals with medium to severe mitral stenosis or mechanical heart valves for stroke prevention in patients with AF [35]. However, there is an absence of data pertaining to individuals suffering from AF and CA and the best anticoagulant therapy, which is why current guidelines recommend anticoagulant selection based on the criteria mentioned above [35].

Cariou et al. revealed that in comparison to patients with DOAC, the results showed that patients with VKA experienced greater bleeding issues (20 vs. 10%; P = 0.013), while there was no difference in stroke occurrences (4 vs. 2%; P = 0.223). Regarding bleeding problems, there was no discernible variation in the subset of AL. The authors speculate that this may be because warfarin-using individuals had worsening renal function, which raised their HAS-BLED hemorrhagic score [45].

Efficacy of VKAs

Acenocoumarol, fluindione, and coumadin are examples of VKAs, which were the initial anticoagulants administered to individuals requiring anticoagulation due to previous episodes of atrial arrhythmia. Bleeding difficulties were more likely among VKA patients [3]. When on anticoagulant medication with VKA, there appears to be an increased risk of death, regardless of the amyloidosis subtype. When renal function and age are considered, this risk disappears. Recent literature also revealed variations in bleeding issues between anticoagulant groups, which were more prevalent among individuals receiving VKA [9]. Due to their decreased renal function, individuals with VKA may have greater HAS-BLED hemorrhagic scores; however, further studies are needed to ascertain this. According to the literature, people with CA are more likely to experience embolic events, and atrial thrombus is more common after transesophageal echocardiography or surgery. We discovered no discernible variations in events between patients receiving VKA and those receiving DOACs, despite our studies confirming this higher risk.

Clinical outcomes

Recent literature reveals that patients who are prescribed VKAs have a higher rate of bleeding as compared to those who used DOACs** **[43,44]. A similar study further highlighted that there were more major bleeds with the use of VKAs as opposed to DOACs. This study went further to differentiate hemorrhagic risk and likelihood of stroke in patients with AL-CA, showing no difference between the two in this type [46]. Cariou et al. were also able to highlight that patients who underwent VKA treatment have an additional risk of mortality, but the same is not established among patients with no renal impairment and younger age groups. A theory posited for this is that patients are less likely to be administered DOACs if they have renal impairments [45]. The enhanced hemorrhagic risk was thought to be due to these patients’ comorbidities, such as renal impairment, resulting in a greater likelihood of bleeding [45].

DOAC in AF

Much research was conducted that reported a high incidence of thrombotic risk in patients with CA who have AF. In one of the research projects that was done on autopsies of patients with CA, it was observed that the prevalence of thrombi in the heart was 33% as compared to the control group [25].

Patients with AF normally receive anticoagulant therapy with respect to their CHA2DS2-VASc score, but recent studies have shown its limited role in correlation with the prevalence of embolic events [25,47]. The left atrial appendage thrombus on a TEE and the CHA2DS2-VASc score appeared to have the least correlation, according to a study of individuals with ATTR-CA and AF [48]. According to the Scientific Statement from the American Heart Association, starting anticoagulation in such patients with CA and AF, irrespective of the CHA2DS2-VASc score, is beneficial [37].

There are two main classes of drugs used as anticoagulants: VKA and DOAC. In the previous scenario, VKA was used as the main anticoagulant, but with recent research, DOAC has been added to European and US guidelines as an alternative anticoagulant [47,49]. The DOACs used today are apixaban, edoxaban, rivaroxaban (factor Xa inhibitor), and dabigatran (factor IIa inhibitor). Patients taking DOAC have a decreased risk of bleeding compared to those getting VKA, according to limited research. A retrospective cohort of 273 patients with CA and a history of AA who required long-term anticoagulation was studied from January 2012 to July 2020. Of these patients, 69 (25%) had AL, 179 (66%) had ATTRwt, and 25 (9%) had variant transthyretin amyloidosis. There was no distinction in stroke occurrences (4 vs. 2%; P = 0.223) between those who got DOAC [45]. In an intention-to-treat analysis, it was reported that patients treated with high-dose edoxaban had a significantly lower risk of annual major bleeding as compared to patients with warfarin (3.43% with warfarin versus 2.75% with high-dose edoxaban, P = 0.01) [42]. In another study comparing rivaroxaban and warfarin, it was reported that the primary endpoint was reached in 269 patients with rivaroxaban and 306 patients with warfarin, with significantly less risk of intracranial (0.5% vs. 0.7%, P = 0.02) and fatal bleeding (0.2% vs. 0.5%, P = 0.003) [41].

In an investigation, Mitrani et al. were unable to demonstrate a statistically noteworthy distinction in severe bleeding among individuals receiving warfarin and DOAC treatment: 5.21 per 100 person-years for DOAC individuals against 3.74 per 100 person-years for VKA patients (P = 0.45) [48]. According to another study by Vilches et al. [7], a total of 531 patients with AF received oral anticoagulation, 322 received VKA, and 239 received DOAC. They informed us that the incident rate of bleeding events with VKA was 3.2 per 100 patient-years (95% CI: 2-5) and 5.1 per 100 patient-years (95% CI 3-8.5), so it did not significantly differ (P = 0.074). Nevertheless, there is limited data on optimal anticoagulation therapy and whether significant differences occur in the bleeding event between VKA and DOAC. All the studies above help us to conclude that the efficacy of DOAC in CA with AF is on par with VKA treatment.

Discussion

The management of anticoagulation in AF associated with CA is a critical aspect of patient care. The option to choose between VKAs and DOACs is a key consideration. The reviewed literature clearly indicates that VKAs and DOACs are both effective in reducing the risk of stroke in AF patients with CA [17,50,51]. VKAs have been the traditional choice for anticoagulation, but DOACs have emerged as a viable alternative with comparable efficacy and safety profiles [52,53]. VKAs require regular monitoring of the international normalized ratio (INR), and patient INR is maintained between 2 and 3. On the other hand, DOACs require a baseline evaluation of complete blood count, glomerular filtration rate (GFR), and liver function tests, with periodic measurement of GFR if the patient is elderly or has a comorbid condition [54]. DOACs and VKAs are considered equally effective and safe in AF patients with CA, but the effectiveness and safety of direct current cardioversion and antiarrhythmic drugs are not well established due to the increased risk of drug toxicity and arrhythmias [55]. In a long-term clinical scenario, DOAC has a lower incidence of stroke, severe hemorrhage, and an all-cause mortality rate when compared to VKAs [56].

Renal function plays a crucial role in the selection of anticoagulants, as impaired renal function can affect the pharmacokinetics and pharmacodynamics of these agents [57,58]. DOACs are primarily excreted via the renal system, hence the need for an individualized approach for patients with varying renal dysfunctions. Use of DOACs is restricted in patients with creatinine clearance <30 mL/min for dabigatran and <15 mL/min for factor Xa inhibitors [57]. For example, rivaroxaban at 15 mg once daily in mild renal dysfunction achieved the same plasma concentration when compared to rivaroxaban at 20 mg once daily with normal renal function [53]. Additionally, patient preference and comorbid conditions, such as concomitant use of nonsteroidal anti-inflammatory drugs (NSAIDs) or aspirin (ASA), should be carefully evaluated when choosing an anticoagulant [59]. A risk versus benefit assessment should be done by the clinician for the concomitant use of anticoagulants and ASA/NSAIDs, as in the case of pericarditis, as anticoagulant use can lead to a higher chance of cardiac tamponade [59]. Use of VKAs requires regular INR monitoring, and initiation requires heparin bridging to prevent an initial hypercoagulable state. A recent study found that VKAs-associated adverse drug reactions, mainly hemorrhagic, were the cause of hospitalizations in the elderly. This was also one of the major reasons why the patients stopped taking VKAs in the first year of initiation [60].

The integration of these findings into current clinical practices emphasizes the need for individualized decision-making when selecting anticoagulation therapy for AF patients with CA. According to Aimo et al., anticoagulation should be started in all individuals with atrial arrhythmias, irrespective of their CHA2DS2-VASc score [57]. In patients with sinus rhythm, when the atria are dilated and proper functioning is affected, anticoagulation will be beneficial, but the bleeding risk of the patient should be considered before prescribing [57]. However, in a study conducted by Kumar and Bhaskaran, patients who had anticoagulation withheld with lower CHA2DS2-VASc scores had more benefits [61]. Clinicians should consider the overall efficacy, safety, and patient-specific factors to optimize the management of anticoagulation in this patient population. The evidence supports the use of both VKAs and DOACs to lower the risk of stroke in AF patients linked with CA, with careful consideration of renal function, patient preference, and potential drug interactions. Drug interaction is identified in cases where both dexamethasone and VKAs are used together for the treatment of CA, which leads to prolonged INR [62]. In CA patients who are predominantly in the older age group, due to coagulopathy and amyloid deposition, blood vessels are brittle, which in turn increases the bleeding tendency in the elderly [25]. Treatment with VKA in this population is challenging because of the inter- and intra-patient heterogeneity as well as dietary and medication compliance. Since all patients on VKA had a labile INR, which is a risk factor for increased bleeding, it is imperative to keep the INR within the normal range [47].

In research conducted by Cariou et al., patients who were on VKA had more bleeding complications when compared to patients with DOAC, but no change in stroke occurrence was identified [45]. These insights confirm the importance of a personalized approach to anticoagulant selection in clinical practice, highlighting the need for ongoing assessment and monitoring to ensure optimal patient outcomes.

Clinical implications and future directions

Patients with CA undergo extensive cardiac remodeling that could result in arrhythmias like AF [63]. This is a significant issue, with up to 70% of CA patients reportedly presenting with this arrhythmia and consequently higher rates of thromboembolism [64]. Over the years, guidelines for the management of AF in CA have failed to recognize and address just how high the prevalence of thromboembolism is in this patient population, with the risk of intracardiac thrombus being as high as 33% even in well-managed patients [64]. The European Society of Cardiology Guidelines propose that CA patients with AF should be initiated on anticoagulation therapy according to the CHA2DS2-VASc risk score [63]. However, the score has proven to be ineffective for CA patients who present with an increased risk of intracardiac thrombus, independent of the CHA2DS2-VASc score [63]. Because the score does not accurately predict a CA patient’s eligibility for anticoagulation therapy, it should not be applied because of the risk of mismanagement in this patient group. This supports the already extensive evidence that CA patients with AF should undergo anticoagulant therapy regardless of their CHA2DS2-VASc risk score [19,63]. This also indicates a need for more research into thromboembolic risk stratification for CA patients. Future studies should be conducted in an effort to develop a new risk assessment score that considers the unique presentation of CA patients when it comes to AF, intracardiac thrombus, and eligibility for anticoagulation therapy.

Ideally, anticoagulant therapy should be tailored to each patient, and a thorough risk-benefit assessment is necessary before initiation to ensure that these medications can be safely administered. Relative contraindications such as kidney dysfunction, underlying bleeding disorders, and potential drug-drug interactions should be carefully assessed, and alternative therapies should be considered [8]. For instance, DOACs like edoxaban and dabigatran can be used for anticoagulation therapy in this patient group; however, 50% and 80% of the medications are excreted by the kidneys, respectively. Hence, kidney function should be closely monitored, as these medications are contraindicated in patients with a creatinine clearance <30 ml.

Alternate therapies like VKAs can be initiated in this subgroup of patients. However, these medications, like DOACs, come with their own risk profile, among which an increase in bleeding time is at the forefront [62]. The bleeding risk is further increased in the presence of a concomitant bleeding disorder such as acquired factor X deficiency, which is common in amyloidosis [63]. Therefore, close monitoring of the INR is recommended, as CA patients taking VKAs have shown significant prolongation of this ratio [8,46,64]. Lastly, drug-drug interaction is an important factor to consider in CA patients because, even in the absence of kidney dysfunction and a bleeding disorder, CA is a multifaceted disease that requires numerous interventions and medications, thus increasing the risk of drug interactions. This can be limited by implementing a multidisciplinary treatment approach in which there is coordination of drug regimens and treatment modalities across different specialties and members of a care team in order to avoid any adverse interactions [64].

## Conclusions

CA is a rare disease with a significant clinical impact. It is associated with atrial arrhythmias, with AF being the most common. Anticoagulation is essential to these patients’ care to lower morbidity and death. The use of VKAs and DOACs is efficacious in stroke prevention; however, a higher bleeding risk is noted in patients who use VKAs. The difference in this bleeding risk was attributed to the clinical profile of the patients who used VKAs, patients with renal impairment, and higher bleeding risks. The CHA2DS2-VASc scoring used to assess patients’ AF stroke risk has a limited role, as it has been highlighted that despite the score, this unique population of patients has a high risk of thrombotic events. It is recommended that a scoring system for patients with CA and AF be utilized to appropriately capture the need for anticoagulation therapy. Most importantly, CA requires a multidisciplinary approach to management due to its complex nature and impact on multiple organ systems, including cardiology, hematology, neurology, nephrology, and radiology.
